# THE ENCORE FELLOWSHIPS PROGRAM: FINDINGS FROM HOST AND FELLOW FOLLOW-UP EVALUATIONS

**DOI:** 10.1093/geroni/igz038.1371

**Published:** 2019-11-08

**Authors:** Cal J Halvorsen, Jim Emerman

**Affiliations:** 1 Boston College School of Social Work, Chestnut Hill, Massachusetts, United States; 2 Encore.org, San Francisco, California, United States

## Abstract

The Encore Fellowships Network (EFN) began as a pilot program in 2009 to match mid- and late-career for-profit employees in the San Francisco Bay Area to non-profit organizations seeking their skills and experience. After a successful pilot, the EFN expanded nationally, reaching 26 states and placing close to 2,000 Fellows in 10 years. This presentation will reveal analysis from more than 1,400 linked surveys of fellows and their organizational hosts both pre- and post-fellowship, totaling more than 350 observations. Results indicate a high sense of program satisfaction and enduring impact on the work of the non-profits. Further, the efficiency of the matching process was positively associated with fellows’ recommendation of the program to friends or colleagues and a higher sense of enduring impact among fellows was positively associated with the likelihood of pursuing post-fellowship non-profit work. Implications for the EFN and the broader nonprofit sector will be discussed.

